# Down-Regulation of Claudin-2 Expression by Cyanidin-3-Glucoside Enhances Sensitivity to Anticancer Drugs in the Spheroid of Human Lung Adenocarcinoma A549 Cells

**DOI:** 10.3390/ijms22020499

**Published:** 2021-01-06

**Authors:** Hiroaki Eguchi, Haruka Matsunaga, Saki Onuma, Yuta Yoshino, Toshiyuki Matsunaga, Akira Ikari

**Affiliations:** 1 Laboratory of Biochemistry, Department of Biopharmaceutical Sciences, Gifu Pharmaceutical University, 1-25-4 Daigaku-nishi, Gifu 501-1196, Japan; 146008@gifu-pu.ac.jp (H.E.); 165077@gifu-pu.ac.jp (H.M.); 165019@gifu-pu.ac.jp (S.O.); yoshino-yu@gifu-pu.ac.jp (Y.Y.); 2 Education Center of Green Pharmaceutical Sciences, Gifu Pharmaceutical University, Gifu 502-8585, Japan; matsunagat@gifu-pu.ac.jp

**Keywords:** chemosensitivity, claudin-2, cyanidin-3-glucoside, lung adenocarcinoma

## Abstract

Claudin-2 (CLDN2), an integral membrane protein located at tight junctions, is abnormally expressed in human lung adenocarcinoma tissues, and is linked to drug resistance in human lung adenocarcinoma A549 cells. CLDN2 may be a target for the prevention of lung adenocarcinoma, but there are few compounds which can reduce CLDN2 expression. We found that cyanidin-3-glucoside (C3G), the anthocyanin with two hydroxyl groups on the B-ring, and cyanidin significantly reduce the protein level of CLDN2 in A549 cells. In contrast, pelargonidin-3-glucoside (P3G), the anthocyanin with one hydroxyl group on the B-ring, had no effect. These results suggest that cyanidin and the hydroxyl group at the 3-position on the B-ring play an important role in the reduction of CLDN2 expression. The phosphorylation of Akt, an activator of CLDN2 expression at the transcriptional level, was inhibited by C3G, but not by P3G. The endocytosis and lysosomal degradation are suggested to be involved in the C3G-induced decrease in CLDN2 protein expression. C3G increased the phosphorylation of p38 and the p38 inhibitor SB203580 rescued the C3G-induced decrease in CLDN2 expression. In addition, SB203580 rescued the protein stability of CLDN2. C3G may reduce CLDN2 expression at the transcriptional and post-translational steps mediated by inhibiting Akt and activating p38, respectively. C3G enhanced the accumulation and cytotoxicity of doxorubicin (DXR) in the spheroid models. The percentages of apoptotic and necrotic cells induced by DXR were increased by C3G. Our data suggest that C3G-rich foods can prevent the chemoresistance of lung adenocarcinoma A549 cells through the reduction of CLDN2 expression.

## 1. Introduction

Cyanidin is an abundant polyphenolic pigment present in vegetation. C3G, known as chrysanthemin, is one of the most widely distributed anthocyanins with glucose bound to the cyanidin at the 3-position on the C-ring. It can be detected in red to blue fruits and vegetables, such as blackberry and black olive [1]. The mechanisms of pharmacokinetics of anthocyanins have been investigated in rodents and humans [2,3]. Anthocyanins are quickly absorbed from the small intestine after oral administration and exist in unchanged forms (glycosides) or metabolized forms (glucuronidated, sulphated, and methylated derivatives). These forms are distributed not only in the digestive system, but also in all other tissues. C3G has potent superoxide radical-scavenging activity, with an IC_50_ of 69.6 µM [4]. It possesses beneficial effects on many diseases, such as diabetes [5] and inflammation [6], possibly due to its antioxidant activity. In addition, C3G has antineoplastic activity against cancer cells, including breast [7], colorectal [8], and esophageal cancer [9]. However, the mechanism of the antitumor activity of C3G is not fully understood yet.

Epithelial cells establish tight junctions (TJs) at the lateral membrane between neighboring cells. The TJs are involved in the regulation of cell proliferation, polarization, differentiation, and polarity [10,11]. In addition, they rigorously regulate the paracellular flux of solutes and ions. Claudins (CLDNs), essential components of the TJs, comprise a large family of over 20 subtypes, and play a role in the barrier function of TJs [12,13]. CLDN2 expressed in leaky epithelia, including proximal tubules of the kidney and small intestine, forms a narrow (∼6.5 Å diameter) and cation-selective pore [14]. We previously reported that the expression level of CLDN2 is increased in human lung adenocarcinoma tissues [15], and is involved in the increased proliferation [16], migration [17], and drug-resistance [18] of human lung adenocarcinoma A549 cells. Therefore, CLDN2 may be a novel target for the development of anticancer drugs. Some flavonoids, including kaempferol, luteolin [19], quercetin [20], and kaempferide [21], can reduce the expression level of CLDN2 in A549 cells. The transcriptional activity of CLDN2 is up-regulated by the phosphoinositide 3-kinase (PI3K)/protein kinase B (Akt) and mitogen-activated protein kinase (MEK)/extracellular signal regulated kinase (ERK) pathways. Kaempferide increases the chemosensitivity of A549 cells to anticancer drugs, mediated by the suppression of the phosphorylation of Akt and CLDN2 expression [21]. However, the effects of C3G on the chemosensitivity and the CLDN2 protein in lung adenocarcinoma cells remain unclear.

In the present study, we examined the effects of C3G, cyanidin and pelargonidin-3-glucoside (P3G) on CLDN2 expression by Western blotting and real-time polymerase chain reaction (PCR) in A549 cells. C3G strongly reduced the protein levels of CLDN2 (over 60%), whereas the mRNA level and promoter activity only slightly decreased (only 30%). Therefore, we investigated the effects of C3G on the destabilization of CLDN2 protein. Furthermore, chemosensitivity to DXR, an anthracycline antibiotic, was assessed using a three-dimensional (3D) spheroid culture model.

## 2. Results

### 2.1. Effects of C3G, P3G, and Cyanidin on the Protein Level of CLDN2 in A549 Cells

**Figure 1:**
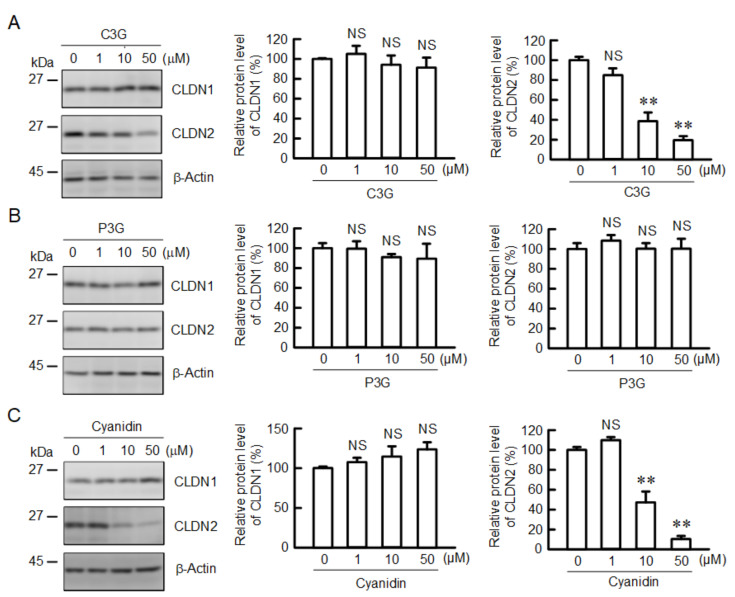
Effects of anthocyanins on the protein levels of CLDN1 and CLDN2 in A549 cells. Cells were incubated with 0, 1, 10, or 50 µM C3G (**A**), P3G (**B**), or cyanidin (**C**) for 24 h. Cell lysates were immunoblotted with anti-CLDN1, anti-CLDN2, and anti-β-actin antibodies. The protein levels of CLDN1 and CLDN2 are represented as a percentage relative to 0 µM. *n* = 3–4. ** *p* < 0.01 and NS *p* > 0.05 compared with 0 µM.

In the search for functional food components, we found that C3G, an anthocyanin, can reduce the protein level of CLDN2 in A549 cells (Figure 1). First, we examined the effects of the hydroxyl group at the 3’-position of the benzene ring (Pelargonidin-3-glucoside, P3G) and carbohydrate removal (cyanidin). The protein level of CLDN2 was reduced by both C3G and cyanidin in a dose-dependent manner, whereas P3G had no effect. The protein level of CLDN1, which is widely expressed in human lung epithelial cells, was unchanged by anthocyanins and cyanidin. This is the first report demonstrating that C3G reduces CLDN2 expression in cancer cells. Therefore, we investigated the mechanism of C3G-induced CLDN2 reduction.

### 2.2. Effect of Anthocyanins on the Cell Localization of CLDN2

**Figure 2:**
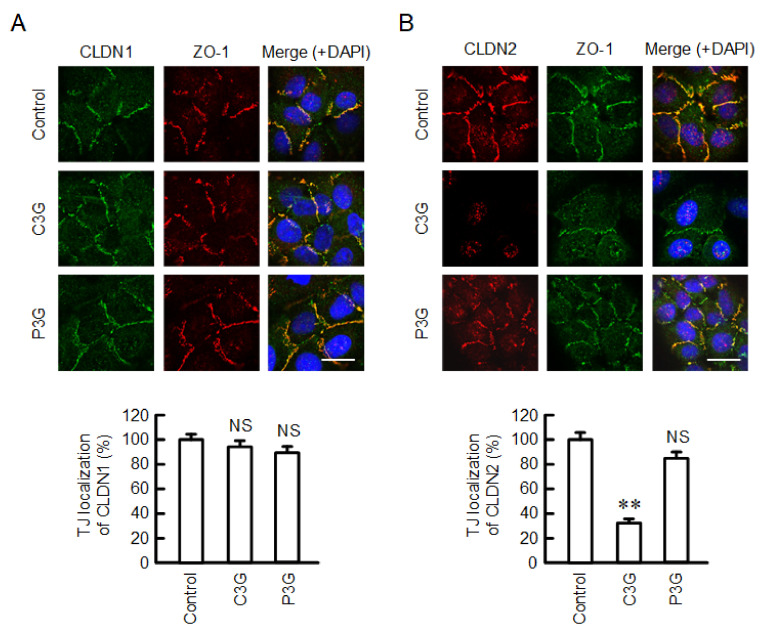
Effects of anthocyanins on the cellular localization of CLDN1 and CLDN2. Cells cultured on cover glasses were incubated in the absence (control) and presence of 50 µM C3G or P3G for 24 h. (**A**) The cells were stained with anti-CLDN1 (green), anti-ZO-1 (red), and nuclear marker DAPI (blue). (**B**) The cells were stained with anti-CLDN2 (red), anti-ZO-1 (green), and DAPI (blue). Merged images are shown in the right panel. The scale bar represents 10 µm. The TJ localizations of CLDN1 and CLDN2 are shown as percentage of control (*n* = 132-220 cells). ** *p* < 0.01 and NS *p* > 0.05 compared with control.

CLDNs are anchored to the actin filaments by binding to zonula occludens-1 (ZO-1), which is an adaptor protein of the TJs [22]. Both CLDN1 and CLDN2 were colocalized with ZO-1 at the cell–cell border area under control conditions (Figure 2). C3G attenuated the fluorescence intensity of CLDN2 without affecting that of CLDN1. In contrast, the localization of CLDN1 or CLDN2 was not significantly changed by P3G.

### 2.3. Effect of Anthocyanins on the mRNA Level of CLDN2

**Figure 3:**
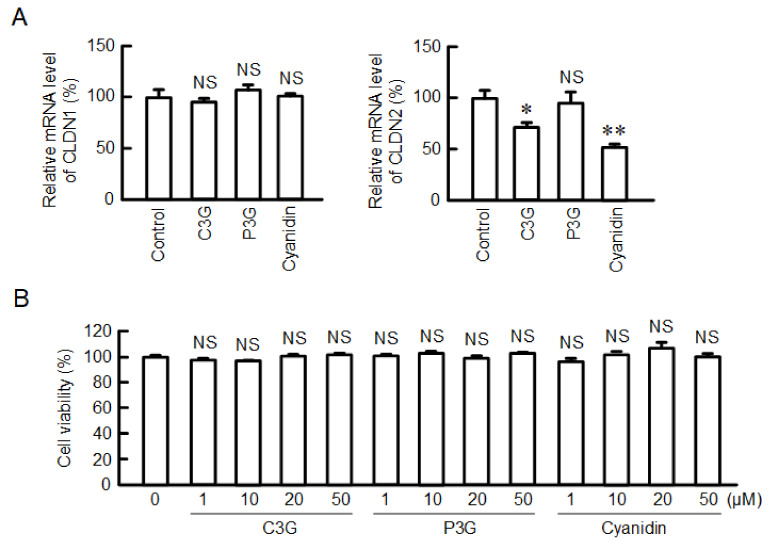
Effects of anthocyanins on the viability and mRNA levels of *CLDN1* and *CLDN2*. (**A**) Cells were incubated in the absence (control) and presence of 50 µM anthocyanins for 6 h. The mRNA levels were measured by quantitative real-time PCR and represented as a percentage relative to control. (**B**) Cells were incubated with 0, 1, 10, 20, or 50 µM anthocyanins for 24 h, followed by the cell viability assays. *n* = 4. ** *p* < 0.01, * *p* < 0.05 and NS *p* > 0.05 compared with control or 0 µM.

The mRNA level of *CLDN2* was significantly reduced by both C3G and cyanidin, but not by P3G (Figure 3A). The mRNA level of *CLDN1* was unchanged by C3G, cyanidin, and P3G. These results are consistent with those of Western blotting. There is a possibility that the cytotoxic effects of C3G and cyanidin are involved in the reduction of CLDN2 expression. However, C3G, P3G, and cyanidin exhibited no significant cytotoxicity up to a concentration of 50 µM (Figure 3B). This indicates that cytotoxicity may not be involved in the reduction of CLDN2 expression by C3G and cyanidin.

### 2.4. Effect of C3G on Intracellular Signaling Pathways

**Figure 4:**
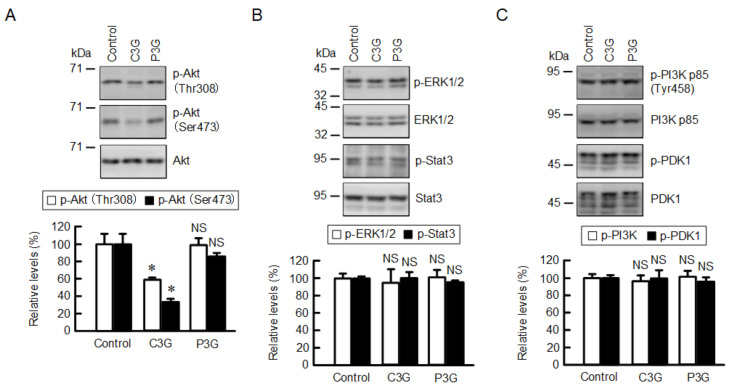
Effects of anthocyanins on intracellular signaling pathways. Cells were incubated in the absence (control) and presence of 50 µM anthocyanins for 1 h. (**A**) Cell lysates were immunoblotted with anti-p-Akt (Thr308), anti-p-Akt (ser473), and anti-Akt antibodies. (**B**) Cell lysates were immunoblotted with anti-p-ERK1/2, anti-ERK1/2, anti-p-Stat3, and anti-Stat3 antibodies. (**C**) Cell lysates were immunoblotted with anti-p-PI3K p85 (Tyr458), and anti-p-PI3K p85, anti-p-PDK1, and anti-PDK1 antibodies. The protein levels were represented as a percentage relative to control. *n* = 4. * *p* < 0.05 and NS *p* > 0.05 compared with control.

We previously reported that the transcriptional activity of CLDN2 is up-regulated by the PI3K/Akt [23], MEK/ERK [15], and Janus kinase (Jak)/signal transducer and activator of transcription (Stat) [19] pathways in A549 cells. The effects of C3G and P3G on the intracellular signaling pathways were examined by Western blotting. C3G reduced the p-Akt level without affecting the total amount of Akt (Figure 4). In contrast, P3G had no effects on p-Akt and Akt levels. Neither the p-ERK1/2 nor the p-Stat3 levels were changed by C3G and P3G. The phosphorylation of Akt is controlled by upstream regulators, phosphoinositide-dependent kinase 1 (PDK1) and PI3K. Neither p-PI3K p85 nor p-PDK1 levels were changed by C3G and P3G. These results indicate that C3G may reduce the protein level of CLDN2 through the suppression of Akt phosphorylation.

### 2.5. Effect of C3G on the Protein Stability and Transcriptional Activity of CLDN2

**Figure 5:**
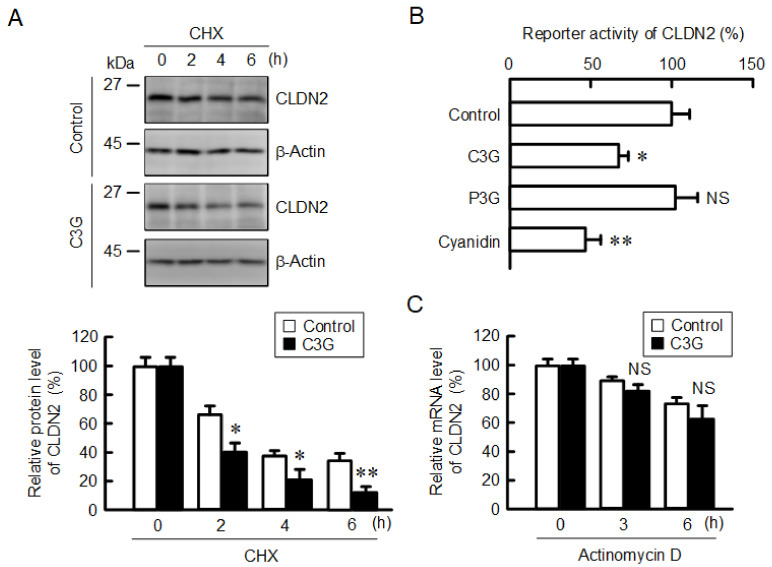
Decrease in the protein stability and reporter activity of CLDN2 by C3G. (**A**) Cells were incubated with 10 µM CHX in the absence and presence of 50 µM C3G for 0, 2, 4, and 6 h. Cell lysates were immunoblotted with anti-CLDN2 and anti-β-actin antibodies. The protein levels of CLDN2 are represented as a percentage relative to control. (**B**) Cells were incubated in the absence (control) and presence of 50 µM anthocyanins for 6 h, and then the reporter activity was measured by the Dual-Luciferase Reporter Assay System. The reporter activity is represented as a percentage relative to control. (**C**) Cells were incubated with 1 µg/mL actinomycin D in the absence and presence of 50 µM C3G for 0, 3, and 6 h. The mRNA level of CLDN2 was measured by quantitative real-time PCR and represented as a percentage relative to 0 h. *n* = 3–5. ** *p* < 0.01, * *p* < 0.05 and NS *p* > 0.05 compared with control or 0 h.

The protein level of CLDN2 was reduced by approximately 80% by 50 µM C3G (Figure 1), but the effect on the mRNA level of *CLDN*2 was lower, approximately 30% (Figure 3A). Therefore, we investigated the effects of C3G on the protein stability of CLDN2. The protein level of CLDN2 time-dependently decreased in the presence of cycloheximide (CHX), a translational inhibitor (Figure 5A). The decrease in CLDN2 was significantly increased by C3G. Next, we examined the effects of anthocyanins on the transcriptional activity and mRNA stability of *CLDN*2. Both C3G and cyanidin reduced the promoter activity of CLDN2, whereas P3G had no effect (Figure 5B). The effect of C3G on promoter activity was approximately 30%, consistent with real-time PCR results. The mRNA level of *CLDN*2 was not significantly decreased by C3G in the presence of actinomycin D, a transcription inhibitor (Figure 5C). These results indicate that C3G may reduce CLDN2 expression through the inhibition of transcriptional activity and the destabilization of protein without affecting mRNA stability.

### 2.6. Effects of Endocytosis, Lysosome and Protein Phosphatase Inhibitors on the C3G-Induced CLDN2 Reduction

**Figure 6:**
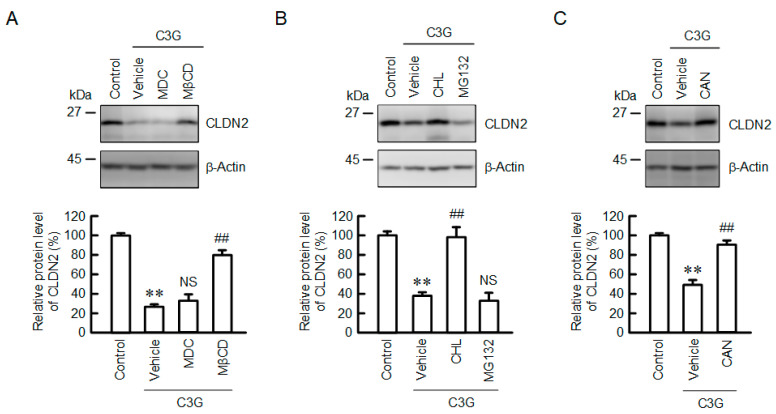
Effect of C3G on the degradation and endocytosis of the CLDN2 protein. (**A**) Cells were incubated in the absence (control) and presence of 50 µM C3G, 5 µM MDC, or 10 µM MβCD for 24 h. (**B**) Cells were incubated in the absence (control) and presence of 50 µM C3G, 20 µM CHL, or 5 µM MG132 for 24 h. (**C**) Cells were incubated in the absence (control) and presence of 50 µM C3G for 24 h. CAN (0.5 µM) was added into the medium 9 h before collection. Cell lysates were immunoblotted with anti-CLDN2 and anti-β-actin antibodies. The protein levels of CLDN2 are represented as a percentage relative to control. *n* = 3–5. ** *p* < 0.01 compared with control. ## *p* < 0.01, and NS *p* > 0.05 compared with vehicle.

To clarify the mechanism of destabilization of CLDN2 protein, we examined the effects of methyl-β-cyclodextrin (MβCD), a caveolae-dependent endocytosis inhibitor, and monodansylcadaverine (MDC), a clathrin-dependent endocytosis inhibitor. The C3G-induced decrease in CLDN2 expression was rescued by MβCD, but not by MDC (Figure 6). In addition, chloroquine (CHL), a lysosome inhibitor, blocked the C3G-induced decrease in CLDN2 expression, whereas MG132, a proteasome inhibitor, had no effect. These results indicate that the acceleration of caveolae-dependent endocytosis and the degradation in the lysosome may be involved in the decrease in CLDN2 expression caused by C3G. The cell localization of CLDN2 is regulated by its phosphorylation status [24]. Cantharidin (CAN), a protein phosphatase 1 and 2A inhibitor, blocked the C3G-induced decrease in CLDN2 expression. There arises the possibility that protein phosphatase is involved in the reduction of CLDN2 by C3G.

### 2.7. Effect of p38 Mitogen-Activated Protein Kinase (MAPK) Inhibitor on the C3G-Induced CLDN2 Reduction

**Figure 7:**
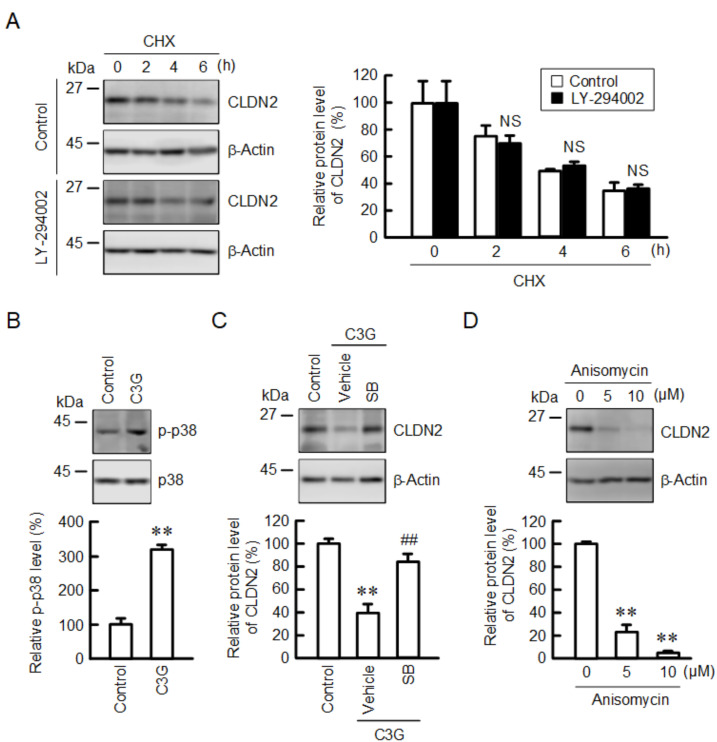
Effect of C3G on the levels of p-p38 and CLDN2. (**A**) Cells were incubated with 10 µM CHX in the absence (control) and presence of 10 µM LY-294002 for 0, 2, 4, and 6 h. Cell lysates were immunoblotted with anti-CLDN2 and anti-β-actin antibodies. The protein levels of CLDN2 are represented as a percentage relative to 0 h. (**B**) Cells were incubated in the absence and presence of 50 µM C3G for 1 h. Cell lysates were immunoblotted with anti-p-p38 and anti-p38 antibodies. The p-p38 levels are represented as a percentage relative to control. (**C**) Cells were incubated in the absence and presence of 50 µM C3G and 10 µM SB203580 (SB) for 24 h. Cell lysates were immunoblotted with anti-CLDN2 and anti-β-actin antibodies. The protein levels are represented as a percentage relative to control. (**D**) Cells were incubated with 0, 5, and 10 µM anisomycin for 24 h. Cell lysates were immunoblotted with anti-CLDN2 and anti-β-actin antibodies. The protein levels are represented as a percentage relative to 0 µM. *n* = 3–4. ** *p* < 0.01 and NS *p* > 0.05 compared with control. ## *p* < 0.01 compared with vehicle.

The effect of LY-294002, an inhibitor of PI3K, on the stability of CLDN2 protein was examined because C3G reduced the p-Akt level. As shown in Figure 5A, C3G promoted the decrease in CLDN2 protein level in the presence of CHX, but LY-294002 did not (Figure 7A). This indicates that C3G may reduce the protein level of CLDN2 through another mechanism different from the PI3K/Akt pathway. C3G increased the p-p38 level without affecting the total amount of p38 (Figure 7B). Furthermore, SB203580, a p38 MAPK inhibitor, blocked the C3G-induced decrease in CLDN2 expression (Figure 7C). Anisomycin, a p38 MAPK activator, dose-dependently reduced the protein level of CLDN2 (Figure 7D). These results support the idea that the p38 MAPK pathway may be involved in the regulation of the protein stabilization of CLDN2.

### 2.8. Effect of p38 MAPK Inhibitor on the Stability of CLDN2 Protein

**Figure 8:**
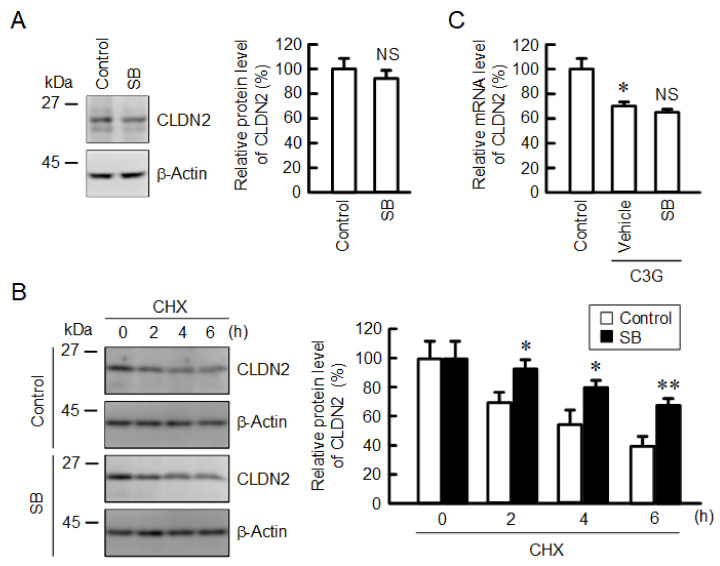
Effects of anisomycin and SB203580 on CLDN2 expression. (**A**) Cells were incubated in the absence (vehicle) and presence of 10 µM SB203580 (SB) for 24 h. Cell lysates were immunoblotted with anti-CLDN2 and anti-β-actin antibodies. The protein levels of CLDN2 are represented as a percentage relative to vehicle. (**B**) Cells were incubated with 10 µM CHX in the absence (control) and presence of 10 µM SB203580 (SB) for 0, 2, 4, and 6 h. The protein levels are represented relative to 0 h. (**C**) Cells were incubated in the absence (control) and presence of 10 µM C3G and 10 µM SB203580 for 6 h. The mRNA levels are represented as a percentage relative to control. *n* = 3–4. ** *p* < 0.01 and * *p* < 0.05 compared with 0 µM or control. NS *p* > 0.05 compared with vehicle.

Next, we examined the effect of SB203580 on the protein stability of CLDN2. The protein level of CLDN2 was not significantly changed by SB203580 (Figure 8A). In contrast, the time-dependent decrease in CLDN2 expression by CHX was attenuated by SB203580 (Figure 8B). The C3G-induced decrease in the mRNA level of *CLDN2* was not rescued by SB203580 (Figure 8C), suggesting that it did not affect the transcriptional activity of CLDN2. Thus, C3G may reduce the stability of the CLDN2 protein through the activation of the p38 MAPK pathway.

### 2.9. Effect of C3G on Transepithelial Electrical Resistance (TER) and Paracellular Permeability

**Figure 9:**
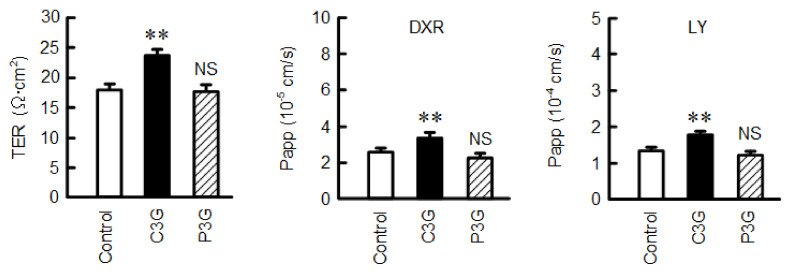
Effect of anthocyanins on the barrier function of TJs. Cells were cultured on transwell inserts and treated with 0 µM (control), 50 µM C3G, or 50 µM P3G for 24 h. TER was measured using a volt-ohmmeter. DXR and LY were applied to the apical compartment. The buffer in the basal compartment was collected after 30 min, and fluorescence intensity was measured. Papp was calculated as described in the method using equation 1. *n* = 3–4. ** *p* < 0.01 and NS *p* > 0.05 compared with control.

CLDN2 forms a paracellular cation channel permeable to Na^+^ and water, and TER is decreased in the CLDN2-expressing cells [25]. C3G significantly increased TER, whereas P3G had no effect (Figure 9). In addition, C3G increased both the transepithelial permeabilities of DXR and lucifer yellow (LY). We previously reported that CLDN2 knockdown significantly increased TER, and increased the paracellular permeability to DXR and LY of A549 cells [18]. Therefore, these results are consistent with those in the CLDN2 knockdown experiments.

### 2.10. Effect of C3G on the DXR-Induced Cytotoxicity in the Spheroid Model

**Figure 10:**
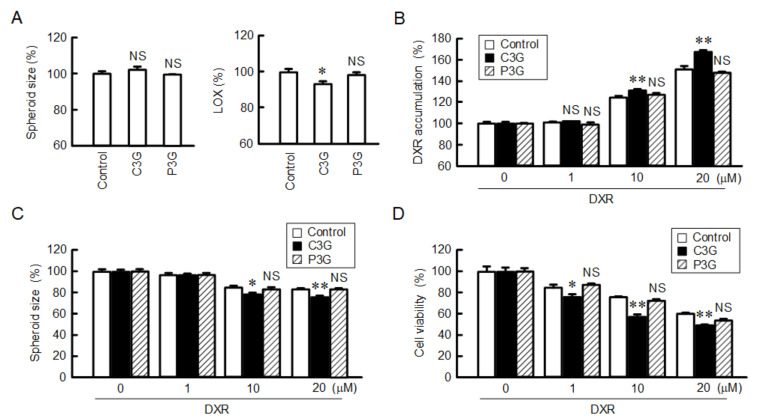
Increase in DXR-induced toxicity by C3G in spheroid cells. Cells cultured on PrimeSurface 96U multi-well plates were treated with 0 µM (control) and 50 µM anthocyanins for 24 h. (**A**) The spheroid size and fluorescence intensity of LOX-1 are represented as a percentage relative to control. (**B**) After treatment with anthocyanins, the cells were incubated with DXR at the indicated concentrations for 1 h. The accumulation of DXR is represented as a percentage relative to 0 µM DXR. (**C**,**D**) The cells were incubated with DXR at the indicated concentrations for 24 h. The spheroid size and cell viability are represented as a percentage relative to 0 µM DXR. *n* = 4–6. ** *p* < 0.01, * *p* < 0.05, and NS *p* > 0.05 compared with control.

The tumor microenvironment plays a role in the chemoresistance of solid tumors. Solid tumors are typically more resistant to chemotherapy than cancer cells cultured as monolayers. In vitro spheroid models can mimic the main features of human solid tumors such as hypoxia, cellular layered assembling and nutrient gradients [26]. We examined the effect of C3G on hypoxia and chemosensitivity using the A549 spheroid model. Neither C3G nor P3G significantly changed the spheroid size (Figure 10A). The hypoxia level was decreased by C3G, but not by P3G. The fluorescence intensity of DXR in the spheroids increased in a dose-dependent manner, which was furthered by C3G (Figure 10B). In addition, DXR reduced the size and viability of the spheroid cells in dose-dependent manners, which were enhanced by C3G (Figure 10C,D). In contrast, P3G did not reduce the cell viability of spheroids. These results indicate that C3G may enhance the chemosensitivity of A549 spheroid cells through the reduction of CLDN2 expression.

### 2.11. Effects of Anthocyanins on Apoptotic and Necrotic Cell Death Induced by DXR

**Figure 11:**
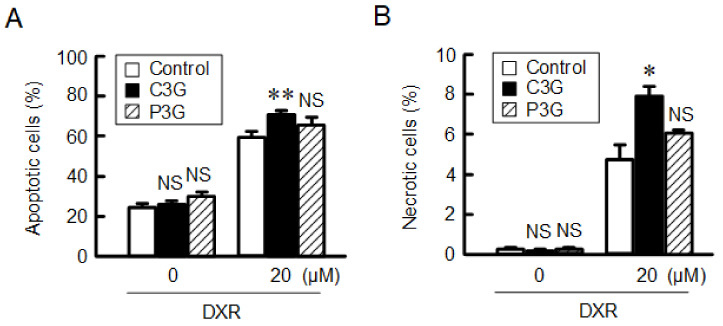
Increase in the rates of DXR-induced apoptotic and necrotic cell death by C3G. After being treated in the absence (control) and presence of 50 µM anthocyanins for 24 h, the spheroid cells were incubated with 0 or 20 µM DXR for 24 h. The rates of apoptotic (**A**) and necrotic cell death (**B**) were examined using a BZ-X800 fluorescence microscope. *n* = 4. ** *p* < 0.01, * *p* < 0.05, and NS *p* > 0.05 compared with control.

Next, we examined the effects of anthocyanins on cell death using an apoptotic/necrotic/healthy cells detection kit. Based on annexin V staining, approximately 20% of cells were apoptotic in the spheroids (Figure 11A). The percentage of apoptotic cells was increased by DXR by approximately 60%, which was increased by C3G. Based on ethidium homodimer-III staining, necrosis was increased by DXR by approximately 5%, which was increased by C3G (Figure 11B). In contrast, P3G did not show significant effects on apoptosis and necrosis. C3G may increase the chemotoxicity by increasing the rate of apoptotic and necrotic cell death in the spheroid model.

## 3. Discussion

The effects of anthocyanins on the CLDN’s expression and epithelial barrier function have been reported using the intestines of animal models. Chronic high-fat diet feeding increases intestinal permeability by reducing the expression of TJ proteins (CLDN1, occludin, and ZO-1), which is rescued by the intake of anthocyanins (glycosides of cyanidin and delphinidin) [27]. Red raspberries rich in anthocyanins reduce CLDN2 protein and increase CLDN3 protein in the dextran sulfate sodium-induced acute colitis model [28]. This is the first report demonstrating that CLDN2 expression is reduced by C3G in lung adenocarcinoma A549 cells. However, we cannot deny the possibility that the effect of C3G may be artefactually specific for A549, because it is not confirmed in other cells. Therefore, it is necessary to clarify the effect of C3G on CLDN2 expression using other lung adenocarcinoma cell lines and/or animal models in a further study. On the other hand, P3G did not have inhibitory effects on CLDN2 expression. The structural difference between C3G and P3G is the presence of the hydroxy group at the 3-position on the B-ring. The hydroxyl groups on the B-rings of flavonoids have an important role in the antioxidant activity [29]. Antioxidant effects are one of the key factors to prevent the development of cancer [30]. However, we previously reported that antioxidant capacity is not involved in the reduction of CLDN2 expression by flavonoids [19]. The hydroxy groups on the B-ring in C3G may play an important role in the reduction of CLDN2 expression independently of antioxidant capacity.

The transcriptional activity of CLDN2 expression is up-regulated by the PI3K/Akt, MEK/ERK, and Jak/Stat3 pathways in A549 cells [15,19,23]. C3G decreased the phosphorylation level of Akt without affecting p-PDK1 or p-PI3K p85 levels, which are upstream signaling mediators of Akt (Figure 4). The molecular mechanism of Akt inhibition by C3G has not been fully understood. We recently reported that chrysin, which has no substituent on the B-ring, can directly bind to Akt [31]. The number and position of hydroxyl groups on the B-ring of flavonoids may play a key role in the inhibition of Akt. In addition, C3G increased the phosphorylation of p38 MAPK (Figure 7B). C3G inhibits the oxidative stress-induced phosphorylation of p38 MAPK in pancreatic β-cells [32] and dermal fibroblast [33]. In contrast, C3G and cyanidin-3 rutinoside increase the phosphorylation level of p38 MAPK in human primary megakaryocytes [34] and leukemic cells [35], respectively. At present, we do not know why C3G has an opposite effect on p38 MAPK. The C3G-induced reduction of CLDN2 protein was inhibited by SB203580, whereas the expression of CLDN2 was reduced by anisomycin, a p38 MAPK activator, in the absence of C3G (Figure 7). These data suggest that C3G may promote the reduction of the CLDN2 protein mediated through the activation of p38 MAPK. However, we cannot deny the possibility that other intracellular signaling factors may be involved in the C3G-induced reduction of the CLDN2 protein, because SB203580 could non-specifically inhibit PDK1 [36] or activate ERK1/2 [37].

The C3G-induced reduction in CLDN2 protein was inhibited by CAN (Figure 6), suggesting that the stability of the CLDN2 protein is controlled by protein kinases or protein phosphatases. In Madin-Darby canine kidney cells, the amount of TJ localization of CLDN2 is increased by its phosphorylation status at Ser208 [24]. Dephosphorylated CLDN2 is translocated from the TJs to the cytosol and then degraded in the lysosome. The C3G-induced decrease in CLDN2 expression was inhibited by MβCD, but not by MDC (Figure 6). These results suggest that a caveolae-dependent pathway may be involved in the internalization of CLDN2. CLDN5 and occludin are internalized via the caveolae-dependent endocytosis pathway in brain endothelial cells [38]. In contrast, CLDN1 and CLDN4 are internalized by the clathrin-dependent pathway in a model of calcium depletion [39]. The endocytic pathways of CLDNs may be different in each subtype and stimuli. Further studies are needed to clarify the relationship between p38 MAPK and the internalization of CLDN2 protein.

Tumor hypoxia promotes malignant progression and chemotherapy resistance [40]. C3G slightly but significantly reduced the hypoxic level without affecting spheroid size (Figure 10A). In addition, C3G enhanced the accumulation and sensitivity to DXR (Figure 10B,D) by increasing apoptotic and necrotic cell death (Figure 11). These results are similar to those of CLDN2 knockdown cells in our previous report [18]. Hypoxic inducible factor-1 expression is up-regulated under hypoxia conditions, and plays a role in the drug resistance of solid tumors via numerous mechanisms, including the induction of drug efflux pumps and drug-metabolizing enzymes [41]. It is unknown how the hypoxic condition of spheroids is remedied by C3G, but C3G may suppress the chemoresistance mediated by the reduction in CLDN2 expression and the enhanced DXR accumulation.

Taken together, C3G may reduce the expression of CLDN2 through both the inhibition of Akt phosphorylation and the promotion of p38 MAPK phosphorylation in A549 cells. C3G reduced the paracellular ion permeability, whereas it increased the transepithelial fluxes of DXR and LY via the inhibition of CLDN2 expression. The sensitivity to DXR was enhanced by C3G in the spheroid model, leading to increased apoptotic and necrotic cell death. Our study suggests C3G to be a useful food component in preventing CLDN2-induced chemotherapy resistance in lung adenocarcinoma A549 cells.

## 4. Materials and Methods

### 4.1. Materials

Rabbit anti-CLDN1 (51-9000) and anti-CLDN2 (51-6100) antibodies were purchased from Thermo Fisher Scientific (Rockford, IL, USA). Rabbit anti-Akt, anti-p-Akt (Ser473), PI3K p85, p-PI3K p85 (Tyr458), and anti-ERK1/2 antibodies were from Cell Signaling Technology (Beverly, MA, USA). Mouse anti-p-Stat3 (pY705), anti-Stat3, and anti-p38 antibodies were from BD Biosciences (San Jose, CA, USA). Rabbit anti-p-Akt (Thr308), anti-p-ERK1/2, and goat anti-β-actin antibodies were from Santa Cruz Biotechnology (Santa Cruz, CA, USA). LY and MDC were from Tokyo Chemical Industry (Chuo-ku, Tokyo, Japan). C3G, CHL, and MβCD were from FUJIFILM Wako Pure Chemical Industries (Chuo-ku, Osaka, Japan). CAN, Cyanidin, mouse anti-ZO-1 antibody, P3G, rabbit anti-p-p38 antibody, and SB203580 were from Cayman Chemical Company (Ann Arbor, MI, USA), Tokiwa Phytochemical (Sakura-shi, Chiba, Japan), Zymed Laboratories (South San Francisco, CA, USA), Toronto Research Chemicals (North York, ON, Canada), Cusabio (Houston, TX, USA), and AdipoGen (San Diego, CA, USA), respectively. All other reagents were of the highest grade of purity available.

### 4.2. Cell Culture

Human lung adenocarcinoma A549 cells were obtained from the RIKEN BRC through the National Bio-Resource Project of the MEXT (Tsukuba-shi, Ibaraki, Japan). The cells were grown in Dulbecco’s modified Eagle’s medium (DMEM, Sigma-Aldrich, St. Louis, MO, USA) supplemented with 5% fetal bovine serum (Biological Industries, Israel), 0.07 mg/mL penicillin-G potassium, and 0.14 mg/mL streptomycin sulfate in a 5% CO_2_ atmosphere at 37 °C. Cell viability in a two-dimensional (2D) culture model was determined by 4-[3-[4-iodophenyl]-2-4(4-nitrophenyl)-2H-5-tetrazolio-1,3-benzene disulfonate] (WST-1) assay.

### 4.3. Sodium Dodecyl Sulfate-polyacrylamide Gel Electrophoresis (SDS-PAGE) and Immunoblotting

Cell lysates containing cell membrane and cytosolic fractions were prepared as described previously [19]. Aliquots of cell lysates (30-80 μg) were applied to SDS-PAGE and blotted onto a poly (vinylidenefluoride) membrane. After incubating with the primary antibody (1:1000 dilution) at 4 °C for 16 h, the membrane was incubated with a horseradish peroxidase-conjugated secondary antibody (1:3000 dilution) at room temperature for 1.5 h. The blots were incubated in EzWestLumi plus (Atto Corporation, Tokyo, Japan) and scanned by a C-DiGit Blot Scanner (LI-COR Biotechnology, Lincoln, NE, USA). Band density was quantified using ImageJ software (National Institute of Health, Bethesda, MD, USA).

### 4.4. RNA Isolation and Quantitative Real-Time PCR

Total RNA was isolated from the cells using TRI reagent (Molecular Research Center, Cincinnati, OH, USA). Reverse transcription and quantitative real-time PCR reactions were performed as described previously [19]. The threshold cycle (Ct) was calculated using the instrument’s software and normalized by subtracting the Ct values of β-actin.

### 4.5. Immunofluorescence Measurement

The tight junctional localizations of CLDN1, CLDN2, and ZO-1 were examined by immunocytochemistry, as described previously [21].

### 4.6. Luciferase Reporter Assay

A549 cells cultured on 24-well plates were transiently transfected with pGL4 luciferase vector (Promega, Madison, WI, USA) containing the 5′-promoter region of human CLDN2 and the pRL-TK vector. The reporter activity of CLDN2 was measured as described previously [15]. The pRL-TK vector was used as an internal control for transient transfections.

### 4.7. TER and Paracellular Permeability

A549 cells were cultured on transwell plates (0.4 μm pore size, 12 mm diameter) with polyester membrane inserts (Corning Incorporated, Corning, NY, USA). The barrier function of TJ was estimated by TER as described previously [42]. An apparent permeability coefficient (Papp) of DXR and LY was calculated as the following equation.

Papp (cm/s) = (dC/dt) × V/C0/A(1)

whereby dC/dt is the change in basal concentration in 30 min (μg/mL/s), *V* is the volume of the chamber, *C*_0_ is the initial concentration of DXR and LY in the apical solution, and A is the area of the chambers (cm^2^).

### 4.8. D Spheroid Model

Cells were cultured on PrimeSurface96U multi-well plates (Sumitomo Bakelite, Tokyo, Japan) for 72 h. The size, hypoxia level, and chemosensitivity of the spheroids were measured as described previously [42]. The percentages of apoptotic and necrotic cells in spheroids were measured using an apoptotic/necrotic/healthy cells detection kit (PromoKine, Heidelberg, Germany).

### 4.9. Statistical Analysis

The results were presented as means ± S.E.M. Differences between groups were analyzed by one-way or two-way analysis of variance. The corrections for multiple comparison were made using Tukey’s multiple comparison test. Comparisons between two groups were analyzed using Student’s *t-*test. Statistical analyses were performed using KaleidaGraph v. 4.5.1 software (Synergy Software, Reading, PA, USA). Significant differences were assumed at *p* < 0.05.

## Data Availability

These data used in this paper is available from public repositories.

## Abbreviations

CAN	Cantharidin
CHL	Chloroquine
CLDN	Claudin
Ct	Threshold cycle
C3G	Cyanidin-3-glucoside
DMEM	Dulbecco’s Modified Eagle’s medium
DXR	Doxorubicin
ERK	Extracellular signal-regulated kinase
FasL	Fas ligand
LY	Lucifer yellow
MAPK	Mitogen-activated protein kinase
MβCD	Methyl-β-cyclodextrin
MDC	Monodansylcadaverine
MEK	Mitogen-activated protein kinase
PCR	Polymerase chain reaction
PDK1	Phosphoinositide-dependent kinase 1
PI3K	Phosphoinositide 3-kinase
P3G	Pelargonidin-3-glucoside
p-Ser	Phosphoserine
p-Thr	Phosphothreonine
SDS-PAGE	SDS-polyacrylamide gel electrophoresis
siRNA	Small interfering RNA
Stat3	Signal transducer and activator of transcription 3
TER	Transepithelial electrical resistance
TJ	Tight junction
WST-1	4-[3-[4-Iodophenyl]-2-4(4-nitrophenyl)-2H-5-tetrazolio-1,3-benzene disulfonate]
ZO-1	Zonula occludens-1
